# A creative collaboration: the arts in psychiatric research

**DOI:** 10.1017/S003329172510233X

**Published:** 2025-10-28

**Authors:** Stefan Priebe, Paul Heritage

**Affiliations:** 1 Centre for Psychosocial Medicine, University of Hamburg, Hamburg, Germany; 2 School of English and Drama, Queen Mary University of London, UK

**Keywords:** Arts, psychiatry, research, collaboration, creativity

## Abstract

There is a rich history of using the arts in psychiatric care, e.g. in various forms of arts therapies. A more recent development is the collaboration of the arts in psychiatric research. The arts and psychiatric research have several fundamental differences which can lead to fruitful inspiration and innovative approaches in research. The benefits include a wider engagement of individuals and non-academic groups in research, creativity in designing research approaches, a reach to societal groups that academic research usually cannot access, and the provision of real life meaning to research evidence. Two examples of such collaborations illustrate the potentials.

Arts and psychiatric research have been linked since psychiatry emerged as a distinct medical and academic discipline in the 19th century. Categorizing their observations of mental distress with terminology borrowed from classical Greek drama, psychiatrists recognized a common purpose in the capacity of artists to explore the human condition and the nature of mental suffering. Just as the work of artists has been interpreted using concepts of psychiatric disorders or theories of psychoanalysis – such as Jung’s model of archetypes or Lacan’s structure of desire – so too has the work of psychiatrists been extended by the incorporation of arts methodologies into their practices. Artwork produced in psychiatric institutions has been propagated by pioneers like Hans Prinzhorn in Germany (Röske, 1995) and Nise da Silveira in Brazil (Kowalski, 2016). Different forms of arts therapies – using modalities such as music, visual arts, drama, and dance – have been established with professional qualifications and manuals for therapists.

In this editorial, we want to outline another and more recent link: the role of arts practices in psychiatric research. Based on our personal experience of collaborating on several – mainly international – research projects over the last 10 years, we consider options for using arts in psychiatric research: the potential benefits and also the problems of such collaborations. We will address the issues in a general way, neither distinguishing between different research methods such as quantitative or qualitative study designs nor between different art forms such as music, theater, or paintings.

The aims and concepts of arts and psychiatric research are fundamentally different. Some of the most important differences are:

- Psychiatric research – like all scientific research – aims to generate insights that are valid for the future and drive the knowledge base forward. Research findings should ideally be ‘true’ and therefore apply now and in the future. Arts consist in the creativity and the experience of the moment: the here and now. What Jane Austen wrote, Shakespeare staged, or Beethoven composed, how Ai Weiwei provokes or Marina Abramovic disrupts may inspire people for centuries, but the experience of the audience – reader, listener, or spectator – is always in the present.
- The findings of research should be generalizable and apply to people beyond the ones who have been investigated in a particular study. The production and experience of the arts can be collective or individual, but there is no claim or requirement that people share the same impression and effects of a piece of art.
- Research produces evidence that is captured in formulae and texts which are fixed, while the arts produce a range of outputs that communicate through written, visual, aural, and sensorial elements that are open to (mis)interpretation
- Research applies methodological rigor and uses transparent and replicable rules, while the arts play with rules and delight in a sleight of hand, a *trompe-l’oiel* or a hidden subtext.
- Research tends to use reductionist thinking with the ideal of objectivity and generalizability, while the practice and experience of the arts is based in subjective truth which might aspire to universality or deny the very concept of truth.

When artists and psychiatric researchers collaborate, they differ in their background and qualifications, their terminology, and often their timescales for working. Even if they both work in an academic setting, the rules and criteria for establishing success and failure also diverge.

These differences can pose a challenge for collaboration, but they can also be the reason for why the arts may stimulate and innovate in psychiatric research. Our experience is that there is a wide interest in the potential of such collaborations, possibly reflecting a general disappointment with the progress achieved through conventional research methods. Further drivers may include an intuitive appeal to the potency of collaborations in arts and psychiatry, mutual respect for the representatives of both fields, an increasing interest from arts organizations in their influence on the mental health of their participants, as well as an increasing general interest in multidisciplinary research in psychiatry.

So, how can arts and psychiatric research collaborate in practice?

- What has already widely happened is that the arts have been an object of research. Psychiatric researchers can explore the impact of arts on mental health in various spheres of society: in education from kindergarten to university, at workplaces, in housing and other built environments, and in social media and online activities.
- The arts offer a way of engaging hard-to-reach participants in research studies. People who may be reluctant to participate in talking groups or individual interviews can be engaged through arts and cultural practices with which they are comfortable and familiar. The arts can be a potent means of accessing feelings, attitudes, ideas, language, behaviors that focus groups, interviews, or surveys tend to miss.
- Arts-based practices may help to identify the most relevant research questions, to find and design the most appropriate methods for studies, and to provide psychiatric researchers with a direction of travel.
- The arts can also be used to engage various stakeholders (such as civil society organizations and non-academic government agencies), to encourage and enable non-academic groups to support and participate in research, and to disseminate the findings to people who may not read scientific publications.
- More importantly, while research tends to produce ‘dry’ evidence, sometimes in forms of numbers and tables, arts may help to create meaning, link these findings with other experiences of people, put them into the context of the real life of people, and explore their potential benefits for changing lives of individuals and communities.

Two examples, from our own collaborations, illustrate the potential contribution of arts-based methods in psychiatric research.

A research program in South America studied factors that help young people living in deprived urban areas to overcome symptoms of anxiety and/or depression (Gomez-Restrepo et al., 2025). The program was conducted with academic partners in Bogotá (Colombia), Buenos Aires (Argentina), and Lima (Peru) using conventional methods such as in-depth interviews, focus groups, a case control study, and a cohort study. In each city, we also invited a cultural organization to collaborate. They were experienced in working with young people using different artistic practices: interactive mixed-arts methods (Crear Vale la Pena, Buenos Aires); training and performance in classical music (Fundacion Batuta, Bogotá); theatre with new writing (La Plaza, Lima). Each organization ran arts workshops with young people to inform the design and selection of the research instruments and support the recruitment of research participants. The successful engagement of participants in these workshops led to the idea of trialing creative theatre workshops for young people with mental distress which were then evaluated with conventional psychiatric research methods (Flores et al., 2025).

In the final year of the program, three young artists from each cultural organization developed workshops/performances to engage young people from their city in the emerging research findings. For example, in Lima, core research findings were first presented and discussed in eight workshops with young people. What the workshop participants said was then – sometimes ad verbatim – taken up by the young artists to create a highly entertaining play called *Gris* (Spanish for ‘gray’) in which the engagement of young people with the city itself was dramatized as a key factor in mental distress (as identified in the research). The play was performed for over a thousand young people in 10 locations across Lima, as well as in Buenos Aires and London. Following the performance, the young artists discussed issues of mental distress including findings of the research program with the audiences in schools, cultural centers, and universities.

The second example is from a research program conducted in India and Pakistan. The program adapted and tested a low-cost generic intervention (DIALOG+) to improve community-based care for people with psychosis (Bird et al., 2023). We supported the academic partners to establish theatre companies made up of people with psychosis, their families/carers, and health professionals. Based in Karachi (Pakistan) and Chennai (India), each theatre company recruited approximately 12 members who were trained in interactive methodologies known as Theatre of the Oppressed, developed in the 1970s by Augusto Boal in Brazil (Boal, 1979). Performed on streets, at festivals and conferences, in crowded hospital waiting rooms and busy offices, with students and their teachers at medical schools, the theatre events gave voice and a new, proactive identity to people with psychosis and those who care for them. It engaged and challenged local communities, medical institutions and general workplaces, opening up conversations and insights about how attitudes – both individual and institutional – severely limit or support community-based care.

In both cases, the arts-based approaches reached and engaged people who traditionally are the subjects of psychiatric research but are normally excluded from process and outputs. Our hope is that research collaborations that value the creative potential of the arts can produce more inclusive, impactful, and human-centered approaches. Will the potential synergy between arts and psychiatry advance our understanding and treatment of mental health? The future of these research practices hinges on delivering benefits that resonate both for the psychiatrists and for the artists that embrace the act of collaboration and ultimately improve mental health care.
